# Compensation models for community health workers: Comparison of legal frameworks across five countries

**DOI:** 10.7189/jogh.11.04010

**Published:** 2021-02-15

**Authors:** Madeleine Ballard, Carey Westgate, Rebecca Alban, Nandini Choudhury, Rehan Adamjee, Ryan Schwarz, Julia Bishop, Meg McLaughlin, David Flood, Karen Finnegan, Ash Rogers, Helen Olsen, Ari Johnson, Daniel Palazuelos, Jennifer Schechter

**Affiliations:** 1 Community Health Impact Coalition, New York, New York, USA; 2 Department of Global Health and Health System Design, Icahn School of Medicine at Mount Sinai, New York, New York, USA; 3 VillageReach, Seattle, Washington, USA; 4 Possible, New York, New York, USA; 5 VITAL Pakistan, Karachi, Pakistan; 6 Division of Global Health Equity, Brigham and Women’s Hospital, Harvard Medical School, Boston, Massachusetts, USA; 7 One to One Africa, Cape Town, South Africa; 8 Medic Mobile, San Francisco, California, USA; 9 Muso, Bamako, Mali; 10 Department of Medicine, Institute for Global Health Sciences, University of California San Francisco, San Francisco, California, USA; 11 Partners In Health, Boston, Massachusetts, USA; 12 Integrate Health, Lome, Togo; 13 THINKMD, Burlington, Vermont, USA; 14 PIVOT, Ranomafana, Madagascar; 15 Wuqu' Kawoq, Santiago Sacatepéquez, Sacatepéquez, Guatemala; 16 Lwala Community Alliance, Rongo, Kenya

## Abstract

**Background:**

Despite the life-saving work they perform, community health workers (CHWs) have long been subject to global debate about their remuneration. There is now, however, an emerging consensus that CHWs should be paid. As the discussion evolves from *whether* to financially remunerate CHWs to *how to do so*, there is an urgent need to better understand the types of CHW payment models and their implications.

**Methods:**

This study examines the legal framework on CHW compensation in five countries: Brazil, Ghana, Nigeria, Rwanda, and South Africa. In order to map the characteristics of each approach, a review of the regulatory framework governing CHW compensation in each country was undertaken. Law firms in each of the five countries were engaged to support the identification and interpretation of relevant legal documents. To guide the search and aid in the creation of uniform country profiles, a standardized set of questions was developed, covering: (i) legal requirements for CHW compensation, (ii) CHW compensation mechanisms, and (iii) CHW legal protections and benefits.

**Results:**

The five countries profiled represent possible archetypes for CHW compensation: Brazil (public), Ghana (volunteer-based), Nigeria (private), Rwanda (cooperatives with performance based incentives) and South Africa (hybrid public/private). Advantages and disadvantages of each model with respect to (i) CHWs, in terms of financial protection, and (ii) the health system, in terms of ease of implementation, are outlined.

**Conclusions:**

While a strong legal framework does not necessarily translate into high-quality implementation of compensation practices, it is the first necessary step. Certain approaches to CHW compensation – particularly public-sector or models with public sector wage floors – best institutionalize recommended CHW protections. Political will and long-term financing often remain challenges; removing ecosystem barriers – such as multilateral and bilateral restrictions on the payment of salaries – can help governments institutionalize CHW payment.

For decades, community health workers (CHWs) – lay workers trained to provide basic health services to their neighbors – have served as a trusted source of primary health care in communities around the world [1]. CHW programs have been widely touted as a means of achieving health for all as early as the Alma-Ata Declaration of 1978 [2]. There is substantial evidence that CHWs can effectively deliver a range of preventive, promotive, and curative health services that ultimately reduce morbidity and mortality, increase access to care, and provide a return on investment of up to 10 to 1 [3,4].

Despite the life-saving work they perform, CHWs have long been subject to global debate about their remuneration. A perception exists among donors and ministry of health officials in many countries that CHW salaries are not ‘sustainable’ [5], that remunerating CHWs might pollute their intrinsic motivation [6], or that CHW-provided services are ‘priceless’ [7]. As such, national cadres of unpaid health workers have not been uncommon over the last forty years [8].

There is now, however, an emerging consensus that CHWs should be paid [9]. The reliance on voluntary CHW work is (i) inconsistent with the international agenda on decent work [10] (including Sustainable Development Goal, SDG, 8 – promoting decent work and economic growth) and (ii) likely to perpetuate gender disparities in access to employment and income opportunities given that the majority of the CHW workforce is female (inconsistent with SDG 5 – achieve gender equality) [11]. Based on these concerns and a systematic review of the evidence, the *2018 WHO Guideline on Health Policy and System Support to Optimize CHW Programmes* strongly recommends remunerating practicing CHWs for their work with a financial package commensurate with the job demands, complexity, number of hours, training and roles that they undertake (Recommendation 7) [12]. This is consistent with the International Labour Organization’s recommendation that pay for health workers “should reflect qualifications, responsibilities, duties and experience” [13]. Since the publication of the WHO guideline, additional evidence has emerged reaffirming the importance of remunerating CHWs consistent with their workload, skills, and responsibilities [14]. Therefore, as the discussion transitions from *whether* to financially remunerate CHWs to *how to do so*, there is an urgent need to better understand the types of CHW payment models and their implications.

This study maps five examples of approaches to CHW payment, considering the ways in which the legal frameworks that underpin these models adhere, or not, to the new WHO guidance on remuneration. Given that the primary health provider in most countries is government, these legal frameworks have wide-reaching implications on health delivery and CHW labour protections, including remuneration. This study intends to serve as a reference point to policymakers, legislators, non-governmental organizations (NGOs), and development agencies, that provide CHWs with financial compensation and employment protections commensurate with the job demands and roles undertaken.

## METHODS

This study examines the legal framework on CHW compensation in five countries: Brazil, Ghana, Nigeria, Rwanda, and South Africa (**Box 1**). Comprehensive case studies of the community health programs in each country are available elsewhere [15]. Countries were selected by members of the Community Health Impact Coalition [16], a network of health practitioners working in over 30 countries that exists to accelerate the uptake of high-impact community health systems design, and financing experts from the Financing Alliance for Health [17] using an online nominal group technique (NGT) [18]. Participants answered the question: ‘based on your professional experience, what grouping of countries might best illustrate a diversity of common approaches to CHW compensation?’

Box 1 **Focus countries and cadres [15].**

**Brazil (agente comunitário de saúde - ACS):** Brazil’s 265 000 ACSs are responsible for providing comprehensive array of promotive, preventive, curative, and rehabilitative services for up to 750 individuals living in a geographically defined area as part of Family Health Teams.

**Ghana (Community Health Volunteer – CHVs):** Ghana’s 19 000+ CHVs support Community Health Officers in providing primary services to up 5000 people in geographic zone.

**Nigeria (Community Health Extension Workers - CHEWs):** Nigeria’s 43 000 CHEWs – one for approximately every 4000 people – provide curative care as extensions of the health service

**Rwanda (Binômes):** Rwanda’s male-female pair of Binômes provide diagnosis and treatment of childhood illnesses, malaria for people of all ages, provision of contraceptives, and TB treatment to villages of approximately 50 to 150 households.

**South Africa (Community Health Workers - CHWs):** South Africa’s 3000-7000+ CHWs provide prevention and promotion, adherence support for chronic lifelong conditions, early identification of ill-health through screening and referral, and basic therapeutic, rehabilitative and palliative care as part Ward-Based Primary Health Care Outreach Teams.

In order to map the characteristics of the approaches, a review of the regulatory framework governing CHW compensation in each country was undertaken. To guide the search and aid in the creation of uniform country profiles, a standardized set of questions was developed (Appendix S1 of the **Online Supplementary Document**). Topics identified included: (i) legal requirements for CHW compensation, (ii) CHW compensation mechanisms, and (iii) CHW legal protections and benefits.

In cooperation with TrustLaw [19], the Thomson Reuters Foundation’s global pro bono legal programme, the researchers identified law firms in each of the five countries willing to apply their expertise of national labor laws and support the identification and interpretation of relevant legal documents. The search took place from December 2019 to May 2020. Documents were included if they were (i) related to community health workers, (ii) a current national policy, law, or regulation, and (iii) the full text was available. For the purposes of this paper, a community health worker was defined as a lay person to whom simple medical procedures can be ‘task shifted’ from more specialized medical providers (eg, nurses, doctors) [20]. In several countries more than one community-based health cadre exists; this paper examines the most formal cadre in each country.

Relevant material was extracted and organized into standardized tables by question to produce a narrative summary of the legal documents [21]. In order to assess the merits and shortcomings of each approach in comparative perspective, the models were marked against the 2018 WHO Guideline and assessed with respect to (i) CHWs, in terms of financial protection, and (ii) the health system, in terms of ease of implementation. The authors provided feedback at various stages of the law firms’ review process in order to promote consistency across the five countries being examined and minimize variability in interpretation by the different law firms involved.

## RESULTS

**Table 1** summarizes each country’s approach to CHW compensation based on several key criteria. Extended country profiles are available in Appendix S2 in the **Online Supplementary Document**.

**Table 1:** CHW compensation models by country

Country (cadre)	Type of compensation model	Payment type	Salary floor / minimum wage	Provision for CHW volunteers	Legal protection
**Brazil (ACS)**	Public sector	Salary	Yes	Yes (through NGOs)	Basic (varies by jurisdiction)
**Ghana (CHVs)**	Volunteer-based	None	No	Yes	None
**Nigeria (CHEWs)**	Private (with minimum wage floor)	Salary	Yes	Yes	Dependent on individual contracts
**Rwanda (Binômes)**	Cooperatives with performance based incentives	Performance-based incentives via cooperatives	No	Yes	None
**South Africa (CHWs)**	Hybrid: Public sector & private (sub-contracting)	Salary (only state-employed CHWs)	Partial	Yes (through NGOs)	Basic (varies by province)

ACS – agente comunitário de saúde, CHW – community health worker, CHEW – community health extension worker, CHV – community health volunteer

### Brazil

**Cadre:** In order to perform the role of a CHW (“agente comunitário de saúde”) in Brazil, an individual must: (i) reside in the area of the community in which they operate; (ii) have successfully completed an initial training course, with a minimum duration of 40 hours; and (iii) have completed high school education [22].

**Legal structure:** Brazil employs a public sector model in which CHWs (“agente comunitário de saúde”) are defined as full-time (40 hours/week) state employees and therefore qualify for a minimum wage that is updated annually [23-25]. CHWs can only be hired directly by the States, Federal District or Municipalities. The temporary or outsourced hiring of CHWs is prohibited except in the event of combating epidemic outbreaks [26].

**Compensation model:** The Federal Constitution and Law No. 11, 350/2006 provide for a national professional salary floor. If CHWs work habitually in unhealthy conditions, as defined by the Ministry of Labour, they are entitled to a health risk premium on top of their base salary [27]. There is a provision for CHWs not employed by the state to act as volunteers, however, this is not a standard practice [28].

**Protections and benefits:** CHWs are generally afforded the same legal protections available to other classes in Brazil. This includes the right to form a union [29].

### Ghana

**Cadre:** CHWs in Ghana (community health volunteer, CHVs) are not subject to certification requirements, although, in practice, they are given informal training by the Ghana Health Service on various aspects of primary health care.

**Legal structure:** Ghana employs a volunteer-based model. Ghana’s Labour Act defines a CHV as a person employed under a contract of employment whether on a continuous, part-time, temporary or casual basis[30]. Ghana’s 2016 Community-based Health Planning and Services Policy (CHPS), however, defines CHVs as non-salaried [31].

**Compensation:** While CHPS states that an appropriate incentives scheme is to be developed and instituted to reward volunteers, CHVs are not considered workers and so do not benefit from a salary floor. There have been proposals from the Ministry of Health in Ghana to retool existing CHVs and regularise the payment system by providing some monetary compensation; these have yet to be formalised and implemented.

**Protections and benefits:** CHVs are not afforded the legal protections available to other classes of workers.

### Nigeria

**Cadre:** CHWs in Nigeria must register with the Community Health Practitioners Registration Board of Nigeria and obtain a certificate prior to practicing [32].

**Legal structure:** Nigeria employs a private model, with a public-sector wage floor. There are no specific laws, regulations or policies on the payment of CHWs. The Labour Act in Nigeria is limited in its scope of application and only regulates the employment of “workers” who are defined as employees who perform manual labour or clerical work. Under Nigerian law, CHWs are classified as Non-Workers (ie, employees who exercise administrative, executive, technical or professional functions) and their compensation is dependent on the terms of their contracts with various employers [33].

**Compensation:** It is an offence for employers to whom the Minimum Wage Act applies to pay less than the specified minimum wage to their employees, including CHWs (N.B. CHW employers could be exempt from the provisions of the Minimum Wage Act if they are (i) an establishment in which workers are employed or paid on part time basis; and commission or piece-rate; (ii) an establishment employing less than 25 persons) [34].

**Protections and benefits:** As non-workers, Nigerian CHWs’ benefits are dependent on the terms of their contracts with their employers [35]. Certain employment-related benefits, such as life insurance and pensions are regulated by Nigerian Law and apply to CHW employment.

### Rwanda

**Cadre:** To become a CHW in Rwanda (“binômes”) one must meet the following requirements: (i) ability to read and write, (ii) aged between 20 and 50, (iii) willing to volunteer, (iv) living in the local village, (v) honest and trusted by the community, and (vi) selected by the village members [36].

**Legal structure:** Rwanda employs a Performance-Based Financing Model centered on Cooperatives. As outlined in Rwanda’s national policies and strategic plans, community health workers are volunteers who receive some compensation according to a performance-based system and income-generating cooperative model [37-39].

**Compensation:** Payment of monetary incentives is dependent upon the CHWs meeting the targets set for each assignment (ie, submit report by the 5th of each month, report completely filled in, etc.). This payment is made directly to cooperatives, which are then tasked with dividing 70% of the money towards income-generating activities, and 30% (plus any revenue) towards cooperative members [40].

**Protections and benefits:** As a result of the volunteer status of CHWs in Rwanda, the majority of legal protections and employment benefits do not apply to CHWs and these are not factored into the CHW compensation program.

### South Africa

**Cadre:** CHWs in South Africa face different requirements depending on whether they are employed in the public sector or subcontracted through NGOs. These are explained below.

**Legal structure:** South Africa employs a Hybrid Public/Private Model. South Africa’s legal framework governing CHW payment is changing, rendering a rating of “partial” for the salary/minimum wage floor criterion [41]. There is no legislation governing the payment of CHWs today. The National Department of Health’s 2018 Policy Framework and Strategy for Ward-Based Primary Healthcare and the 2011 provincial guidelines for primary health care, however, provide a strategic framework for the employment of CHWs by provincial and district departments of health.

**Compensation:** In light of the provisions of this policy framework, the Department of Health concluded an agreement with unions representing CHWs to standardize the remuneration of state-employed CHWs at the same level as the legislated minimum wage [42,43]. These developments apply only to CHWs employed by the State and who meet specific criteria. While certain provinces directly employ CHWs in their respective departments of health, others use NGOs as intermediaries and certain provinces make use of payroll management companies contracted by the health departments to employ CHWs.

**Protections and benefits:** The definitions of “employee” in the core labour legislation in South Africa include persons who “in any manner” assists with or furthers the business of an employer [44]. Thus, all CHWs should be afforded the same protection as any other employees under this legislation. CHWs employed through intermediaries are not protected by the remuneration agreement concluded with the Department of Health, however, and as such remain excluded from the narrower protection of the National Minimum Wage Act of 2018.

## DISCUSSION

This study examines the legal framework on CHW compensation in five countries representing possible archetypes for CHW compensation: Brazil (public), Ghana (volunteer-based), Nigeria (private), Rwanda (cooperatives with performance based incentives) and South Africa (hybrid public/private). Approaches were assessed in terms of legal structures and requirements for CHW compensation, CHW compensation mechanisms, and CHW protections and benefits.

From these data, it is possible to draw preliminary conclusions about potential benefits and pitfalls of each model in terms of operationalizing the WHO guidelines for CHW compensation and the international agenda on decent work (CHWs are recognized in the International Labor Organization’s International Standard Classification of Occupations, ISCO, as a distinct occupational group - ISCO 3253).

The 2018 WHO Guideline on Health Policy and System Support to Optimize Community Health Worker Programmes recommends [45]:

1. “Remunerating CHWs with a financial package commensurate with the job demands, complexity, manner of hours, training and roles that they undertake”; and
2. “Not paying CHWs exclusively or predominately according to performance-based incentives.”

**Table 2** summarizes the way in which the compensation models described above comply or do not comply with the Guideline.

**Table 2:** Assessment of country compliance with WHO recommendation [46]

Country (cadre)	CHW hours	CHW job demands*	CHW remuneration	Meets guideline?
**Brazil (ACS)**	Full time (40 h/week)	1200 h of training	Guaranteed minimum wage & extensive worker protections	**Yes**
				Financial package
		Provide promotive, preventive, curative, and rehabilitative services		Aligned with hours & job demands
**Ghana (CHVs)**	Considered “part time”	40 h of training	None	**No**
	On call 24 h a day, every day [47]	Disease surveillance, health promotion, home management of minor ailments, referrals, transportation, community mobilization		No financial package despite considerable responsibilities, appreciable hours, & rigorous on-call requirements
**Nigeria (CHEWs)**	Full time (40 h/week) [48]	3 y of training	Minimum wage	Yes
				Financial package
		Curative services, referral, health promotion		Aligned with hours & job demands
**Rwanda (Binômes)**	Part time (average of 9 h/week on CHW & cooperative activities) [49]	480 h of training (average)	Performance Based Financing via cooperatives	**No**
				CHWs paid exclusively according to performance based incentives
		Diagnosis and treatment of (esp. child) illness, screening and referral, provision of contraceptives	2/3rds of cooperatives did not make profit & were not able to give dividends to members [49]	Funds may be insufficient in comparison to CHW needs and efforts.
**South Africa (CHWs)**	20-40 h/week (hours are highly variable across the country)	12 mo of training (variable)	Remuneration levels are highly variable across the country, in many instances, below the national minimum wage	**Partial**
		Prevention and promotion, adherence support for chronic lifelong conditions, screening, referral, and basic palliative care		Financial package
				High variability of remuneration for CHWs with similar job demands

ACS – agente comunitário de saúde, CHW – community health worker, CHEW – community health extension worker, CHV – community health volunteer

*Including complexity, training, roles.

Potential lessons for other countries with respect to (i) CHWs, in terms of financial protection, and (ii) the health system, in terms of ease of implementation, are summarized in **Table 3** and **Table 4**. (NB, At the time of writing, compensation for South Africa’s CHWs does not fully comply with the WHO Guidelines. This may, however, be changing. The country is in the process of establishing a minimum wage requirement for state-employed CHWs via a collective bargaining agreement.)

**Table 3:** Models meeting the WHO guideline

Remuneration model	Summary	Advantages	Disadvantages
**Public sector (Brazil)**	CHWs can be hired by states, federal districts, or municipalities.	**CHW:** Provides protections and employment benefits. Requirements of the legislation leaves little room for exploitation.	**CHWs & health system:** Brazilian law imposes several conditions that must be met when hiring CHWs, which does not make this type of hiring flexible.
	Pay for state-employed CHWs adheres to professional salary floor.	**Health system:** Compensation of CHWs is regulated by specific legislation, providing clarity.	
**Private – with public sector wage floor (Nigeria)**	CHWs are regarded as ‘non-workers’ under Nigerian labour legislation and as such, their remuneration is determined by their employment contract. This remuneration, however, must not be below the minimum wage established by the National Minimum Wage Act, 2019.	**CHWs:** Flexibility to negotiate desired terms with their contractors while ensuring that their compensation can never be lower than the national minimum wage.	**CHWs**: May lack negotiating power relative to employers. Lack of government intervention provides an opportunity for exploitation. For instance, CHWs are required to pay an annual fee to retain their ability to practice but are not offered much protection by the government associations.
	CHWs must register with national body and obtain certificate to practice.	**Health system:** The terms of engagement for a CHW are governed by the employment contract, and as a result, the employer may choose to offer additional allowances and incentives.	**Health system:** Potential lack of accountability.

CHW – community health worker

**Table 4:** Models not or partially meeting the WHO guideline

Remuneration model	Summary	Advantages	Disadvantages
**Volunteer-based** (Ghana)	CHVs are unpaid.	**CHWs:** Opportunity to gain experience.	**CHWs:** Few legal protections and/or benefits. Possibility of exploitation.
		**Health system:** No up-front expenditure.	**Health system:** Potential lack of accountability and control.
**Cooperatives with performance based incentives** (Rwanda)	CHWs are legally treated as volunteers and thus not entitled to salary.	**CHW:** Cooperative model provides opportunities to generate income from alternative sources.	**CHWs:** Few legal protections and/or employment benefits. Funds may be insufficient in comparison to CHW needs and efforts; one analysis found more than half reported that lack of financial support was a main obstacle for being effective in their jobs. Possible mismanagement of cooperative operations due to lack of support. Asks CHWs to split their attention between the cooperative enterprise and their job delivering health care.
	Compensation is via performance-based mechanisms plus income-generating cooperative model. Payment is dependent upon the CHWs meeting the targets set for each assignment. This payment is never made directly to individual CHWs but rather to their cooperatives.	**Health system:** No up-front expenditure, and allows for flexibility in terms of budgeting.	**Health system**: While Rwanda’s cooperative model is said to promote financial independence of CHWs and build entrepreneurial skills of CHWs, evidence of this is lacking. Only 1/3 of cooperatives are profitable.
**Hybrid: Public sector & private sub-contracting** (South Africa)	CHWs employed in the public sector or subcontracted through NGOs.		**CHWs & health ystem:** The absence of a single model leads to inequalities between CHWs and inconsistent integration of CHWs into the broader health system.
		**CHWs:** Public sector CHWs are more integrated in the provinces’ systematic responses and have greater work security.	**CHWs:** Existing protections apply only to government CHWs.
	CHWs in the public sector are more likely to be regulated under and benefit from minimum wage legislation and union-negotiated compensation agreements.	**Health system:** Flexibility for non-state actors and competitive landscape. The sub-contracting model relieves provincial health departments from having to manage an additional workforce.	**Health system:** Weak oversight and accountability mechanisms for gov’t sub-contractors managing CHWs: it is far more difficult to regulate work expectations, provision of essential equipment, compensation and remuneration where there is no cohesive structure of employment.

CHW – community health worker, CHV – community health volunteer

### Cross-cutting considerations

#### Volunteerism

The legal structure in every country in-scope for this review includes provisions for engaging community health workers as volunteers, but only some countries mandate volunteerism in their official policies and strategy documents. As noted in the WHO Guideline, the continued existence of dedicated volunteers is not ruled out by the recommendation, though it may be difficult to delineate between willing volunteers (who, eg, have a different full-time job) and those without an alternative source of livelihood whose “choice” is structured by economic insecurity [12,50]. Attention is needed to ensure volunteers supported by non-state actors do so of their own volition and that their workload is commensurate with a volunteer position. It is inherently coercive to ask individuals to volunteer as a condition to access health care for themselves, their family, and their community.

#### Non-state actors

Non-state actors play a large role in managing the activities of community health workers [51]. It is important to include these non-state actors, as well as the bilateral, multilateral, and private philanthropic institutions who help finance community health services, in discussions around formalizing the pay and legal protections for these different cadres of CHWs.

Governments do not make policy in a vacuum; the axis of praise or blame ought not to be laid solely on them. The policies, approaches, and investments of key partners create an ecosystem that can make it either easier or more difficult for a government to take action. Inadequate and restrictive development assistance from bilateral and multilateral institutions, for example, make it difficult for governments to pay CHWs, and in turn the CHWs bear the brunt of these policies [52]. Progress is possible when partners support government leaders in overcoming such challenges and governments step up to renegotiate power relations that no longer serve them.

#### Financing

Implementing the WHO recommendations into law requires both political will and technical/financial resources, which will derive from both international and domestic sources. As indicated by the WHO Guideline, countries “should consider the financial package to remunerate CHWs as a part of the overall health system planning, and adequate resources should be made available” [12]. The Guideline indicates that the government is to mobilize and prioritize the required resources.

On the point of mobilization, it is worth noting that the costs of development, including those related to building health systems, have never been solely borne by the beneficiaries – neither in Europe during the Industrial Revolution nor today [53]. Any concept of ‘sustainable financing’ ought to reflect this reality: international institutions ought to revisit their investments to support legislative frameworks and long-term financing pathways for paid, professionalized CHWs in line with WHO recommendations. Contributing actively to those financing pathways, by removing restrictions that prevent funds from being used on CHW salaries and by investing actively in the costs of paying CHWs, is an important step.

### Limitations

(i) In several countries more than one community-based health cadre exists. This paper was an attempt to examine a diversity of common approaches to CHW compensation. In some cases, results would vary considerably if another cadre were chosen. For instance, an analysis of the Volunteer Village Health Workers cadre in Nigeria, rather than the CHEWs, would likely return results similar to those presented for Ghana’s CHVs [15]. (ii) Design involving several examples of each approach would have made for a stronger foundation to arrive at conclusions. Nonetheless, these initial observations come at a critical time and help lay the groundwork for subsequent inquiry. (ii) A strong legal framework does not necessarily translate into high-quality implementation on the ground. Further research is needed to assess the fidelity with which the legal framework and protections are carried out in communities.

## CONCLUSION

While a strong legal framework for CHW compensation does not necessarily translate into high-quality implementation, it is the first necessary step. The WHO Guideline, referencing the international agenda on decent work, is clear about the bare minimum required. While certain approaches to CHW compensation – particularly public-sector or models with public sector wage floors – best institutionalized WHO recommendations in this analysis, political will and long-term financing often remain obstacles. Removing ecosystem barriers to the passing of necessary legislation – such as multilateral and bilateral restrictions on the payment of salaries – can help governments achieve the necessary protections for CHWs.

## Additional material

Online Supplementary Document
