# Supplementary material for: Compensation models for community health workers: Comparison of legal frameworks across five countries
Source: J Glob Health. 2021 Feb 15;11:04010. doi: 10.7189/jogh.11.04010 (PMC7916445; doi:10.7189/jogh.11.04010)
Supplement: Online Supplementary Document [file jogh-11-04010-s001.pdf]

Appendix S1. Questions for Standardized Country Profiles

Jurisdiction Information

**Country**	

Questions

**Legal Structure and Requirements for CHW Compensation**			
**Question**		**Summary**	**Legal Basis**
1	Are there existing policies, laws, or regulations governing the payment of CHWs?		
2	Is payment determined in relation to national reference standards such as “lowest-paid-government-worker (LPGW)” or in relation to national or international thresholds or poverty lines?		
3	Does CHW compensation vary by region/state, or are CHWs paid allowances for work in “special conditions” like rural/remote postings?		
4	Are there legal prerequisites to be paid as a CHW (e.g. having to undergo licensing or training, etc.)?		

**CHW Compensation Model**

Question		Summary	Legal Basis
5	What is the basic legal structure of the compensation model?		
6	Are CHWs paid through a national insurance programme or otherwise?		
7	Is it illegal to not pay CHWs?		
8	Are there any restrictions on using volunteers to work as CHWs?		

Legal Protections and Benefits for CHWs			
**Question**		**Summary**	**Legal Basis**
9	Do labour laws offer the same legal protections to CHWs as are afforded to other government employees (including, e.g., the ability to form or join a union for collective bargaining, or the ability to protest or organize a strike)?		

10	Are employment-related benefits (e.g. cost-of-living adjustments or pension scheme contributions) and employment requirements (e.g. vaccination) regulated or specified in contracts?		

Appendix S2. Complete Country Profiles

1. BRAZIL

Legal Structure and Requirements for CHW Compensation		
**Summary**		**Legal Basis**
1.	**Are there existing policies, laws, or regulations governing the payment of CHWs?**<br><br>Yes. Article 198, paragraph 5, of the Federal Constitution and Law No. 11,350/2006, provides for the national professional salary floor of the CHW (*"agente comunitário de saúde")*.<br><br>Ordinance No. 2,488/2011 governs the National Policy on Primary Care and sets out the guidelines and norms for the organisation of Primary Care, for the ESF and the PACS. It also provides the structure and the responsibility of the remuneration of CHWs.	http://www.planalto.gov.br/ccivil_03/Constituicao/Constituicao.htm<br><br>http://www.planalto.gov.br/ccivil_03/_Ato2004-2006/2006/Lei/L11350.htm<br><br>http://bvsms.saude.gov.br/bvs/saudelegis/gm/2011/prt2488_21_10_2011.html
2.	**Is payment determined in relation to national reference standards such as "lowest-paid-government-worker (LPGW)" or in relation to national or international thresholds or poverty lines?**<br><br>Article 9-A, 1st paragraph, of Law No. 11,350/2006, establishes the national professional salary floor that the Federal Government, the States, the Federal District and the Municipalities must pay to the CHWs, for working 40 weekly hours. The current national professional salary floor is BRL1,400.00 (as of January 1, 2020). The future amount will be: BRL1,550.00 as from 1st January 2021.[1] The salary floor will be adjusted annually on 1st January.	http://www.planalto.gov.br/ccivil_03/_Ato2004-2006/2006/Lei/L11350.htm
3.	**Does CHW compensation vary by region/state, or are CHWs paid allowances for work in "special conditions" like rural/remote postings?**<br><br>No; the compensation scheme is national, i.e. based on the national professional salary floor. According to Article 9-A, paragraph 3, of Law No. 11,350/2006, habitual and permanent work in unhealthy conditions that exceed the tolerance limits established by the Labour Ministry will entitle the CHWs to a health risk premium, which will be calculated on their salary or base salary. In the case of a CHW whose employment is subject to the legal regime established by the CLT, the additional amount shall be 40% (maximum level), 20% (medium level) or 10% (minimum level) of the minimum wage of the region. If the employment is subject to a work regulation of another nature, the compensation amount will comply with the provisions of	http://www.planalto.gov.br/ccivil_03/_Ato2004-2006/2006/Lei/L11350.htm<br><br>http://www.planalto.gov.br/ccivil_03/decreto-lei/del5452.htm

[1] Approximately US$ 325.00 and US$ 360.00, respectively.

Legal Structure and Requirements for CHW Compensation		
	that specific legislation.	
4.	**Are there legal prerequisites to be paid as a CHW (e.g. having to undergo licensing or training, etc.)?**<br>According to article 6 of Law No. 11,350/2006, the individuals must meet the following requirements to perform the role of a CHW:<br>(i) reside in the area of the community in which they operate, from the date of publication of the public selection procedure notice;<br>(ii) have successfully completed an initial training course, with a minimum duration of 40 hours; and<br>(iii) have completed high school education. When there is no registered candidate who meets the requirement set forth in item (iii), the hiring of a candidate that has completed elementary school may be permitted. This individual must, however, complete high school within a maximum period of 3 years.<br><br>According to article 9 of Law No. 11,350/2006, the hiring of CHWs must be preceded by a public selection process (i) of examinations (ii) or tests (examination and titles (diplomas and certificates attesting to the applicant's educational and vocational training). These tests will be set according to the nature and complexity of the proposed duties and will assess the specific requirements for the exercise of the activities, which must meet the principles of legality, impersonality, morality, publicity and efficiency.	http://www.planalto.gov.br/ccivil_03/_Ato2004-2006/2006/Lei/L11350.htm

CHW Compensation Model		
**Summary**		**Legal Basis**
5.	**What is the basic legal structure of the compensation model?**<br><br>As stated in article 8 of Law No. 11,350/2006, CHWs that are employed by local managers of the SUS and the FUNASA, are subject to the legal regime established by the CLT, with the exception of the States, the Federal District and Municipalities, where local law provides otherwise.	http://www.planalto.gov.br/ccivil_03/_Ato2004-2006/2006/Lei/L11350.htm<br><br>http://www.planalto.gov.br/ccivil_03/decreto-lei/del5452.htm
6.	**Are CHWs paid through a national insurance programme or otherwise?**<br><br>According to Article 2 of Constitutional Amendment No. 51/2006, the CHWs can only be hired directly by the States, Federal District or Municipalities, observing the limit of	http://www.planalto.gov.br/ccivil_03/constituicao/emendas/emc/emc51.htm

CHW Compensation Model		
	expenditure established in complementary law.	
7.	**Is it illegal to not pay CHWs?**<br><br>Law No. 11,350/2006 establishes the national professional salary floor for the CHWs. It would, therefore, be considered illegal for the State, Federal District or Municipality not to pay the CHW or to refuse to observe the national professional salary floor.	http://www.planalto.gov.br/ccivil_03/_Ato2004-2006/2006/Lei/L11350.htm
8.	**Are there any restrictions on using volunteers to work as CHWs?**<br>According to Brazilian law, volunteer service is considered to be the unpaid work done by an individual for a public entity of any nature or to a non-profit private institution that has civic, cultural, educational, scientific, recreational or personal assistance purposes. There is no restriction on using volunteers to work as CHWs as long as the unpaid service is provided to one of the above-mentioned entities, and all other legal requirements are met.<br><br>CHWs can only be hired directly by the States, Federal District or Municipalities, and the current legislation establishes a national professional salary floor for these professionals.<br><br>Additionally, article 16 of Law No. 11,350/2006 prohibits the temporary or outsourced hiring of CHWs except in the event of combating epidemic outbreaks, in accordance with the applicable law.<br><br>Notwithstanding the above-mentioned provisions, Law No. 11,350/2006 does not expressly prohibit voluntary work as volunteers could be used by the States, Federal District or Municipalities in the development of the inherent activities of CHW. However, this is not a standard practice, so any other structure should be analysed individually.	http://www.planalto.gov.br/ccivil_03/leis/l9608.htm<br><br>http://www.planalto.gov.br/ccivil_03/_Ato2004-2006/2006/Lei/L11350.htm

Legal Protections and Benefits for CHWs		
Remarks		Legal Basis
9.	**Do labour laws offer the same legal protections to CHWs as are afforded to other government employees (including, e.g., the ability to form or join a union for collective bargaining, or the ability to protest or organize a strike)?**	http://www.planalto.gov.br/ccivil_03/Constituicao/Constituicao.htm<br><br>http://www.planalto.gov.br/ccivil

Legal Protections and Benefits for CHWs		
	Yes. These legal protections are for all CHWs, regardless of the legal regime to which the CHW is subject, i.e. (i) the CLT; or (ii) the State, Federal District or Municipal Law.	_03/decreto-lei/del5452.htm<br><br>http://www.planalto.gov.br/ccivil_03/_Ato2004-2006/2006/Lei/L11350.htm
10.	**Are employment-related benefits (e.g. cost-of-living adjustments or pension scheme contributions) and employment requirements (e.g. vaccination) regulated or specified in contracts?**<br><br>The minimum labour rights and benefits to be observed will depend on the legal regime to which the employment of CHW is subject, i.e. either (i) the CLT; or (ii) the State, Federal District or Municipal Law.<br><br>The parties (State, Federal District or Municipality and CHW) may establish other benefits and conditions in the employment contract of the CHW, as long as they do not derogate from those provided for in the applicable law.	http://www.planalto.gov.br/ccivil_03/Constituicao/Constituicao.htm<br><br>http://www.planalto.gov.br/ccivil_03/decreto-lei/del5452.htm<br><br>http://www.planalto.gov.br/ccivil_03/_Ato2004-2006/2006/Lei/L11350.htm

2. GHANA

Legal Structure and Requirements for CHW Compensation		
Remarks		Legal Basis
1.	**Are there existing policies, laws, or regulations governing the payment of CHWs?**<br><br>The Government of Ghana through the National Community based Health Planning and Services Policy (CHPS) which was published in March 2016 recognises the role of community health volunteers. The services of CHVs are purely voluntary, and they are non-salaried. The CHPS states that an appropriate incentive scheme is to be developed and instituted to reward volunteers depending on performance, duration of stay and category of deprivation of the CHPS zone they operate in.	https://www.ghanahealthservice.org/downloads/MOH_CHPS_Policy_Final.pdf<br><br>http://fairwages.gov.gh/wp-content/uploads/2018/09/Government-white-paper.pdf
2.	**Is payment determined in relation to national reference standards such as (LPGW) or in relation to national or international thresholds or poverty lines?)**<br><br>Although there is a national daily minimum wage, which is currently GHS11.82 (approximately US$2.21), this applies to workers as defined by the Labour Act, 2013 (Act 651), and does not apply to CHVs.	https://ghanaemployers.com.gh/wp-content/uploads/2019/09/Scanned-Documents-1.pdf
3.	**Does CHW compensation vary by region/state, or are**	

Legal Structure and Requirements for CHW Compensation		
	**CHWs paid allowances for work in "special conditions" like rural/remote postings?**<br><br>CHVs are currently non-salaried, although the CHPS states that an appropriate incentives scheme is to be developed and instituted to reward volunteers.	
4.	**Are there legal prerequisites to be paid as a CHW (e.g. having to undergo licensing or training, etc.)?**<br><br>There are no legal requirements applicable to CHVs, in practice however, such volunteer CHWs are given informal training by the Ghana Health Service. They are trained in various aspects of primary health so that they can help improve access to low-cost essential drugs and services.	

CHW Compensation Model		
Remarks		Legal Basis
5	**What is the basic legal structure of the compensation model?**<br><br>N/A	
6	**Are CHWs paid through a national insurance programme or otherwise?**<br><br>N/A	
7	**Is it illegal to not pay CHWs?**<br><br>The Labour Act requires employers to pay the agreed remuneration at the time and place agreed on in the contact of employment or collective bargaining agreement or by law or agreed between the employer and the worker. However, since CHVs are not workers, there is no legal requirement to pay them.	http://www.melr.gov.gh/wp-content/uploads/2015/11/LABOUR-ACT-2003.pdf
8	**Are there any restrictions on using volunteers to work as CHWs?**<br><br>No.	

Legal Protections and Benefits for CHWs		
Remarks		Legal Basis
9.	**Do labour laws offer the same legal protections to CHWs as are afforded to other government employees (including, e.g., the ability to form or join a union for collective bargaining, or the ability to protest or organize a strike)?**	http://www.melr.gov.gh/wp-content/uploads/2015/11/LABOUR-ACT-2003.pdf

Legal Protections and Benefits for CHWs		
	Section 1 of the Labour Act 2003 (Act 651) provides that the Act applies to all workers and all employers except the Armed Forces, Police Service, the Prison Service, and the Security and Intelligence Agencies specified under the Security and Intelligence Agencies Act 1996 (Act 526).<br><br>CHVs are not workers as per the definition of same in the Labour Act and are therefore not afforded the protections under the Act.	
10.	**Are employment-related benefits (e.g. cost-of-living adjustments or pension scheme contributions) and employment requirements (e.g. vaccination) regulated or specified in contracts?**<br><br>Employment-related benefits such as pension scheme contributions are regulated under the National Pensions Act, 2008 (Act 766).<br><br>The Fair Wages Commission established under the Fair Wages Act, 2007 (Ac 737) is mandated to plan, manage, and direct salary administration under the Single Spine Pay Policy. Benefits such as cost-of-living adjustments are, therefore, regulated under the Fair Wages Act. These benefits do not apply to CHVs.	https://www.ssnit.org.gh/wp-content/uploads/2015/12/Law-Pension-ACT-766.pdf<br><br>http://fairwages.gov.gh/wp-content/uploads/2018/09/The-Act-of-the-Commision.pdf

3. NIGERIA

Legal Structure and Requirements for CHW Compensation		
**Remarks**		**Legal Basis**
1.	**Are there existing policies, laws, or regulations governing the payment of CHWs?**<br><br>There are no specific Nigerian policies, laws, or regulations on the payment of CHWs.<br><br>By way of background, the principal law that regulates labour and employment in Nigeria is the Labour Act, Chapter L1, Laws of the Federation of Nigeria 2004 (the "Labour Act"), as well as the principles of Nigerian case law. The Labour Act is limited in its scope of application and only regulates the employment of "Workers" who are defined in the Labour Act as employees who perform manual labour or clerical work. The relationship between an employer and an employee who is not a "Worker" (such as employees who exercise administrative, executive, technical or professional functions – 'non-worker") is, therefore, primarily regulated by the relevant contract of employment and the principles of Nigerian case law.<br><br>In view of the foregoing, CHWs would be classified as non-	https://lawsofnigeria.placng.org/laws/L1.pdf

	workers and the provisions of the Nigerian Labour Act would not apply to them. Therefore, the terms and conditions of their service will be governed by their respective contracts of employment.	
2.	**Is payment determined in relation to national reference standards such as "lowest-paid-government-worker (LPGW)" or in relation to national or international thresholds or poverty lines?)**<br><br>The Federal Government recently increased the minimum wage for employees in the federal civil service from NGN18,000[2] per month to NGN30,000[3] per month by the National Minimum Wage Act, 2019. It is an offence for employers, to whom the Minimum Wage Act applies, to pay less than the specified minimum wage to their employees.<br><br>The following categories of employers are exempt from the provisions of the Minimum Wage Act:<br><br>(a) an establishment in which workers are employed or paid on –<br>(i) part time basis; and<br>(ii) commission or piece-rate;<br>(b) an establishment employing less than 25 persons;<br>(c) workers in seasonal employment such as agriculture; and<br>(d) persons employed in a vessel or aircraft to which the laws regulating merchant shipping or civil aviation.	http://placbillstrack.org/8th/upload/National%20Minimum%20Wage%20Act,%202019.pdf,
3.	**Does CHW compensation vary by region/state, or are CHWs paid allowances for work in "special conditions" like rural/remote postings?**<br><br>Although the compensation payable to CHWs will depend on their individual contracts of employment, in practice, this would vary by region or state where the CHW is employed. Some employers may also elect to provide additional allowances, subject to the employment contract between the parties.	N/A

[2] Approximately US$50.
[3] Approximately US$80.

Legal Structure and Requirements for CHW Compensation		
4.	**Are there legal prerequisites to be paid as a CHW (e.g. having to undergo licensing or training, etc.)?**<br><br>The Community Health Practitioners (Registration, etc.) Act ("CHPA") requires an individual who wishes to practice as a CHW to register with the Community Health Practitioners Registration Board of Nigeria and obtain a certificate.<br><br>An individual will be entitled to register under the CHPA if:<br>a) The individual has attended a course of training approved by the Community Health Practitioners Registration Board of Nigeria ("Board");<br>(a) the course was conducted at an approved institution(s);<br>(b) the individual holds a Certificate of the National Institute or any equivalent diploma or qualification and has complied with the other requirements prescribed under the CHPA Act;<br>(c) the individual is of good character; and<br>(d) the individual pays any prescribed fee.<br><br>In addition, a person registered under the CHPA is required to pay an annual prescribed retention fee to retain their registration and any failure to pay the retention fee will result in temporary withdrawal of the certificate to practice.<br><br>The Community Health Practitioners Registration Board of Nigeria in collaboration with the National Primary Health Care Development Agency have issued the "National Standing Orders for Community Health Workers/Community Health Extension Workers" to ensure that quality of care at the primary health care level is uniform across the country and all community health workers are required to adhere to the standing orders<br><br>The Federal Ministry of Health has also issued a Manual for the Training of Community Health Extension Worker on LARC Methods (Manual on LARC Training). In order for CHWs to administer injectable contraceptives in communities and LARC methods to women, they are required to undergo this specialised training.	https://www.lawyard.ng/wp-content/uploads/2016/01/COMMUNITY-HEALTH-PRACTITIONERS-REGISTRATION-ETC.-ACT.pdf<br><br>http://nphcda.gov.ng/publications/<br>Manual on LARC Training

CHW Compensation Model		
Remarks		Legal Basis
5.	**What is the basic legal structure of the compensation model?**<br><br>N/A	

CHW Compensation Model		
6.	**Are CHWs paid through a national insurance programme or otherwise?**	
7.	**Is it illegal to not pay CHWs?**<br><br>There is no legal requirement to pay CHWs where there is no existing contract between the CHW and an employer, which requires the employer to make payments to the CHW. A breach of the employer's payment obligations under the contract would be grounds for a civil suit for breach of contract at the NIC.	
8.	**Are there any restrictions on using volunteers to work as CHWs?**<br><br>There does not appear to be any restrictions under Nigerian law on using volunteers to work as CHWs, provided that such volunteers are registered to act in such capacity under the CHPA.	

Legal Protections and Benefits for CHWs		
Remarks		Legal Basis
9.	**Do labour laws offer the same legal protections to CHWs as are afforded to other government employees (including, e.g., the ability to form or join a union for collective bargaining, or the ability to protest or organize a strike)?**<br><br>CHWs are entitled to the same legal protections afforded to other government and non-government employees under Nigerian Law. As such, by virtue of the Trade Unions Act, chapter T14, LFN 2004 as amended by the Trade Union (Amendment) Act 2005, CHWs are not precluded from joining a trade union or from forming a trade union, where no union currently exists for this sector.	

Legal Protections and Benefits for CHWs		
10.	**Are employment-related benefits (e.g. cost-of-living adjustments or pension scheme contributions) and employment requirements (e.g. vaccination) regulated or specified in contracts?**<br><br>CHWs are classified as non-workers under Nigerian law and as a result, their benefits are determined by the relevant contract of employment.<br><br>Certain employment-related benefits, however, such as life insurance and pensions are regulated by Nigerian Law and will apply to the employment of CHWs. The Pension Reform Act, (PRA) 2014 applies to every employer, that has three or more employees. It requires every employer to contribute 10% (ten percent) of an employee's total monthly emolument, to deduct 8% (eight percent) of the employee's monthly emoluments (as the employee's contribution), and to remit the total contribution (18%) into the employee's retirement saving account with a pension fund administrator of the employee's choice within 7 (seven) working days from the date on which its employees' salaries are paid.<br><br>The PRA 2014 also imposes an obligation on employers that have three or more employees to obtain life insurance cover for all their employees, which should be of a value that is at least three times the annual emoluments of each employee.<br><br>Section 48 of the National Health Insurance Scheme Act, Chapter N42 LFN 2004 ("NHIS Act 2004") and section 1.1.1.1 of the NHIS Operational Guidelines 2012 prescribe that employers who have a minimum of ten employees are obliged to enrol themselves and their employees in the National Health Insurance Scheme and pay contributions into the account of a designated health maintenance organisation that is accredited by the NHIS.<br><br>The CHWs may be entitled to additional benefits, if, so provided in their terms of employment. These benefits are:<br>(a) Maternity / Paternity Leave<br>(b) Bereavement/ Compassionate Leave;<br>(c) Leave of Absence;<br>(d) Study Leave;<br>(e) Casual Leave;	https://www.pencom.gov.ng/wp-content/uploads/2017/04/1448643400_PRA_2014.pdf<br><br>http://www.nigeria-law.org/National%20Health%20Insurance%20Scheme%20Decree.htm<br><br>https://drive.google.com/file/d/0B1DAmtM1BcbMS215ci1tOVBSZjg/view

Legal Protections and Benefits for CHWs		
	(f) Payment of 13th Month Salary; and<br>(g) Bonus payments.	

4. RWANDA

Legal Structure and Requirements for CHW Compensation		
**Remarks**		**Legal Basis**
1.	**Are there existing policies, laws, or regulations governing the payment of CHWs?**<br><br>There is a National Community Health Policy (2015) that recognizes CHWs and their remuneration. Section 2.6.1 (Paragraphs 7 and 9) of The National Community Health Policy details the purpose of and challenges facing performance-based financing for CHWs as well as the structure and internal working of the CHW cooperatives.<br><br>The Ministry of Health: National Community Health Strategic Plan (July 2013-June 2018), Pages 9 and 31 indicate that CHWs generally work on a volunteer basis except where compensation is received upon the achievement of pre-determined indicators.	http://moh.gov.rw/fileadmin/templates/policies/NATIONAL_COMMUNITY_HEALTH_POLICY.pdf<br><br>http://www.moh.gov.rw/fileadmin/templates/CHD_Docs/CHD-Strategic_plan.pdf
2.	**Is payment determined in relation to national reference standards such as LPGW or in relation to national or international thresholds or poverty lines?**<br>Payment is not determined in relation to national reference standards such as LGPW nor is it determined in relation to national or international thresholds or poverty lines.	http://moh.gov.rw/fileadmin/templates/policies/Health_Sector_Policy___19th_January_2015.pdf
3.	**Does CHW compensation vary by region/state, or are CHWs paid allowances for work in "special conditions" like rural/remote postings?**<br><br>It does not vary because of region; rather, any variation may be due to the nature of tasks assigned and performance of those tasks.	N/A
4.	**Are there legal prerequisites to be paid as a CHW (e.g. having to undergo licensing or training, etc.)?**<br><br>Trainings are sometimes required. However, this does not apply to all the instances concerning CHWs and their remuneration. Pages 52 and 53 of the National Community Health Strategic Plan 2013 -2018 cover all the instances where	http://www.moh.gov.rw/fileadmin/templates/CHD_Docs/CHD-Strategic_plan.pdf

Legal Structure and Requirements for CHW Compensation		
	training will be required. Trainings are usually required in the following sectors:<br>1) Maternal and childcare health;<br>2) Nutrition or home-based food fortification; and<br>3) Training on management and accountability (for the cooperatives mainly).	

CHW Compensation Model		
Remarks		Legal Basis
5.	**What is the basic legal structure of the compensation model?**<br><br>The compensation model for CHWs includes compensation based on performance from government, returns from CHW cooperatives and other in-kind compensation as is apparent in:<br>i. Pages 23, 26 and 27 of the Health sector policy 2015.<br>ii. Page 4 of The National Community Health Strategic Plan July 2013 – June 2018; and<br>iii. Section 2.6.1 of the National Community Health Policy, 2015	http://moh.gov.rw/fileadmin/templates/policies/Health_Sector_Policy___19th_January_2015.pdf<br><br>http://www.moh.gov.rw/fileadmin/templates/CHD_Docs/CHD-Strategic_plan.pdf<br><br>http://moh.gov.rw/fileadmin/templates/policies/NATIONAL_COMMUNITY_HEALTH_POLICY.pdf
6.	**Are CHWs paid through a national insurance programme or otherwise?**<br><br>The health insurance scheme is one of the sources of funding for their payment.<br><br>However, it is not specifically designed or created to cover payment for CHWs.	N/A
7.	**Is it illegal to not pay CHWs?**<br><br>Pages 51 - 53 of the National Community health strategic plan 2013 -2018 outlines the Minimum Package for Community Health Workers. The CHWs are considered to be volunteers therefore payment is not considered mandatory. However, certain fees are payable for expenses such as transportation, which can be as low as 3000rwf per month [4]	http://moh.gov.rw/fileadmin/templates/policies/Health_Sector_Policy___19th_January_2015.pdf<br><br>http://www.moh.gov.rw/fileadmin/templates/CHD_Docs/CHD-Strategic_plan.pdf
8.	**Are there any restrictions on using volunteers to work as CHWs?**<br>There are no restrictions on using volunteers. CHW Central's	http://www.chwcentral.org/sites/default/files/Rwanda%20-%20Rwanda%27s%20Communit

[4]Approximately US$3.18.

CHW Compensation Model		
	'Rwanda Community Health Worker Program' states that in order to be volunteer as a CHW, a person must meet the following conditions:<br>1) they must be able to read;<br>2) they should be aged between 20 – 50 years;<br>3) they should be willing to volunteer;<br>4) they should be living in that local village in which they are to work;<br>5) they must be trusted by the community members; and<br>6) they must be elected by the community.	y%20Health%20Worker%20Program.pdf

CHW Compensation Model		
Remarks		Legal Basis
9	**Do labour laws offer the same legal protections to CHWs as are afforded to other government employees (including, e.g., the ability to form or join a union for collective bargaining, or the ability to protest or organize a strike)?**<br>CHWs are not afforded similar legal protections to other government employees under the current labour laws. This is because they are mainly seen as volunteers.	
10.	**Are employment-related benefits (e.g. cost-of-living adjustments or pension scheme contributions) and employment requirements (e.g. vaccination) regulated or specified in contracts?**<br>No, they are not included for CHWs. The arrangement is usually that of compensation for work done.	N/A

5. SOUTH AFRICA

Legal Structure and Requirements for CHW Compensation		
Remarks		Legal Basis
1.	**Are there existing policies, laws, or regulations governing the payment of CHWs?**<br>South Africa has no binding laws governing payment of CHWs. However, two government policies offer guidelines for their remuneration, and the National Department of Health and unions representing CHWs in the public sector have entered into a collective agreement for a minimum wage.<br><br>Firstly, the Policy Framework and Strategy for Ward-Based Primary Healthcare Outreach Teams 2018/19 – 2023/24 published in 2018 (the "2018 Policy Framework") aims to ensure appropriate implementation and management of remuneration for CHWs.<br><br>Following the 2018 Policy Framework, and also in 2018, the Public Health and Social Development Sectoral Bargaining Council (the "Bargaining Council") reached an agreement with the Department of	https://rhap.org.za/wp-content/uploads/2018/04/Policy-WBPHCOT-4-April-2018-1.pdf<br><br>https://www.phsdsbc.org.za/wp-content/uploads/2018/06/RESOLUTION-1-OF-2018-AGREEMENT-ON-THE-STANDARDISATION-OF-REMUNERATION-FOR-COMMUNITY-HEALTH-WORKERS-IN-THE-

	Health in terms of which CHWs will receive a non-service remuneration payment of ZAR 3,500[5] per month. This was called the Public Health and Social Development Sectorial Bargaining Council Resolution 1 of 2018: Agreement on the Standardisation of Remuneration for Community Health Workers in the Department of Health (the "Bargaining Council Resolution"). As we detail below, this would fall within the current national minimum wage.<br><br>In 2019, the Minister of Finance allocated ZAR 4.4 billion to CHWs over the course of three years. The allocation forms part of a ring-fenced HIV/AIDS and TB grant. The first CHW allocation of ZAR 1 billion will, however, only be available in the 2021/2022 financial year[6].<br><br>Unless renewed, the Bargaining Council Resolution will expire in April 2020, well before the money is available to provinces for payment of CHWs[7].<br><br>The Bargaining Council Resolution covers public sector CHWs with the requisite school leaving certificate (known as a "matric" or grade 12 certificate) or experience falling under the Recognition of Prior Learning programme.<br><br>The compensation structures described above would therefore exclude many CHWs employed by non-profit organisations, including all registered CHWs in the Western Cape, Northern Cape, Free State, Limpopo (that is, four out of the nine provinces in South Africa) and segments of the registered CHWs in other provinces.	DEPARTMENT-OF-HEALTH.pdf<br><br>The 2018 Policy Framework at Section 5.4, Objective 3 on page 18.
2.	**Is payment determined in relation to national reference standards such as LPGW or in relation to national or international thresholds or poverty lines?**<br><br>The national minimum wage was generally considered not to be applicable to CHWs, who, prior to the Bargaining Council Resolution, would fall within the exclusion of volunteers not entitled to be remunerated. However, we consider the sectoral determination through the Bargaining Council Resolution to "entitle" CHWs employed by the Department of Health to a wage in the manner envisioned by the National Minimum Wage Act 9 of 2018 ("NMW Act"). We therefore consider that CHWs in the public sector must be paid above the national minimum wage. We note, however, that most CHWs appear to be paid below the minimum wage.	NMW Act at section 3(2) https://www.gov.za/sites/default/files/gcis_document/201811/42060gon1303act9of2018.pdf<br><br>See table of provincial programmes compiled in November 2015 by the South African health publication *Spotlight*. The table is described and accessible through: https://www.spotlightnsp.co.za/2015/11/30/3-history-chws-south-africa/
3.	**Does CHW compensation vary by region/state, or are CHWs paid allowances for work in "special conditions"**	https://www.dropbox.com/s/e7dqm9kryh65s3q/Provincial%20Gui

[5] Approximately US$190.
[6] See National Treasury *Budget Review 2019* (20 February 2019) at 58.
[7] See clause 5.4. We understand the final signature to have been made in April 2019.

Legal Structure and Requirements for CHW Compensation		
	**like rural/remote postings?**<br><br>CHW compensation varies by province because each province and district can implement guidelines according to its own objectives and budgets according to the 2011 Provincial Guidelines for the Implementation of the Three Streams of the PHC re-Engineering ("2011 Provincial Guidelines"). The 2018 Policy Framework also allocates responsibilities between national, provincial and district levels. It notes the concept of "rural-proofing", through which the different needs of rural areas and CHWs working in them would be taken into account, but it goes no further than listing it as a term in the glossary.<br><br>The 2018 Policy Framework goes on to allocate to provinces the responsibility of ensuring that the rural context is explicitly considered in implementation. This is appropriate considering provinces have greater insight to needs of specific areas given the variation in socio-economic conditions across the country. It is not clear whether references to the rural context are intended to encourage additional compensation.	delines%20for%20the%20implementation%20of%20the%20three%20Streams%20of%20PHC%20Re-engineering.pdf?dl=0<br><br>Policy Framework and Strategy for Ward-Based Primary Healthcare Outreach Teams 2018/19 – 2023/24
4.	**Are there legal prerequisites to be paid as a CHW (e.g. having to undergo licensing or training, etc.)?**<br><br>Provision 5.1 of the Bargaining Council Resolution records that the agreed payment to CHWs "should be for those CHWs that have matric or either [sic] experience and those that can benefit from RPL will be processed through the personal payment system."<br><br>The 2018 Policy states that CHWs who do not have matric certificates (grade 12 school leavers' certificate) will be either placed or phased out during a transition phase.	https://www.phsdsbc.org.za/wp-content/uploads/2018/06/RESOLUTION-1-OF-2018-AGREEMENT-ON-THE-STANDARDISATION-OF-REMUNERATION-FOR-COMMUNITY-HEALTH-WORKERS-IN-THE-DEPARTMENT-OF-HEALTH.pdf<br><br>Policy Framework and Strategy for Ward-Based Primary Healthcare Outreach Teams 2018/19 – 2023/24 , Section 4.3 at Page 15.

CHW Compensation Model		
Remarks		Legal Basis
5.	**What is the basic legal structure of the compensation model?**<br><br>The National Department of Health's 2018 Policy Framework and 2011 Provincial Guidelines provide a strategic framework	https://www.dropbox.com/s/e7dqm9kryh65s3q/Provincial%20Guidelines%20for%20the%20implementation%20of%20the%20three%20Streams%20of%20PHC%2

	for provincial and district departments of health. The implementation of the framework falls within the concurrent jurisdiction of the National Department of Health and the provincial departments. Roles and responsibilities of implementation are allocated at a national, provincial and district level.<br><br>It is a district level responsibility to ensure that finance is available for required human resources, equipment and process implementation.<br><br>Although there is a guideline, provincial departments have very varied methods for remuneration of CHWs. Certain provinces employ CHWs in their respective departments of health, others use NGOs as intermediaries and certain provinces make use of payroll management companies contracted by the departments to employ CHWs.	0Re-engineering.pdf?dl=0<br><br>Policy Framework and Strategy for Ward-Based Primary Healthcare Outreach Teams 2018/19 – 2023/24<br><br>National Health Act 61 of 2003, Chapter 5 https://www.gov.za/sites/default/files/gcis_document/201409/a61-03.pdf
6.	**Are CHWs paid through a national insurance programme or otherwise?**<br><br>CHWs are paid by provincial departments of health, NGOs or through payroll management companies contracted by the Department of Health to employ CHWs.<br><br>The National Health Insurance Bill, 2018, envisages remunerating health workers, including CHWs, through the National Health Insurance Fund. The Bill is currently in its second iteration. It is highly controversial and its timeframes for enactment at this stage are unclear.	National Health Insurance Bill B-11 2019<br><br>https://www.gov.za/sites/default/files/gcis_document/201908/national-health-insurance-bill-b-11-2019.pdf
7.	**Is it illegal to not pay CHWs?**<br><br>Since the Bargaining Council Resolution, the Department of Health is obliged to ensure that CHWs are compensated under the terms of that resolution. Failure to compensate would amount to a breach of that legal obligation. It does not, however, appear that the Bargaining Council Resolution binds NGOs. In certain circumstances NGOs might be entitled to treat CHWs as volunteers and compensate them only for work-related expenses. (Clause 5.1 of the Bargaining Council Resolution)	https://www.phsdsbc.org.za/wp-content/uploads/2018/06/RESOLUTION-1-OF-2018-AGREEMENT-ON-THE-STANDARDISATION-OF-REMUNERATION-FOR-COMMUNITY-HEALTH-WORKERS-IN-THE-DEPARTMENT-OF-HEALTH.pdf<br><br>Policy Framework and Strategy

CHW Compensation Model		
		for Ward-Based Primary Healthcare Outreach Teams 2018/19 – 2023/24
8.	**Are there any restrictions on using volunteers to work as CHWs?**<br><br>The Department of Health views CHWs as falling somewhere between employees and volunteers.[8] However, all CHWs working directly for the state are required to be treated as employees because of the Bargaining Council Resolution. As far as we are aware, there is no restriction on NGOs using volunteers as CHWs.	N/A

Legal Protections and Benefits for CHWs		
Remarks		Legal Basis
9.	**Do labour laws offer the same legal protections to CHWs as are afforded to other government employees (including, e.g., the ability to form or join a union for collective bargaining, or the ability to protest or organize a strike)?**<br><br>CHWs are entitled to the same legal protections afforded to other government and non-government employees under South African Law. Section 23 of the Constitution gives "everyone" the right to fair labour practices, the right to form and join a trade union and the right to strike.<br><br>The Labour Relations Act (LRA), Basic Conditions of Employment Act (BCEA), Employment Equity Act 55 of 1998 (EEA) and the Occupational Health and Safety Act 85 of 1993 (OHSA) apply to CHWs unless specifically excluded from certain provisions (such as the minimum wage provisions).<br><br>In November 2016, CHWs formed the NUCWOSA in order to unify the struggle for recognition of CHWs. Many CHWs are also members of a broader union called the NUPSAW.<br><br>Labour legislation applicable to CHWs<br>Several provisions of the Constitution of the Republic of South Africa, 1996, are relevant to the employment and equitable remuneration of CHWs. The most important for our purposes	https://www.justice.gov.za/legislation/constitution/SAConstitution-web-eng.pdf<br><br>https://www.wylie.co.za/wp-content/uploads/LABOUR-RELATIONS-ACT-NO.-66-OF-1995.pdf<br><br>https://www.saica.co.za/Portals/0/Technical/LegalAndGovernance/Basic%20Conditions%20of%20Employment%20Act%2075%20of%201997.pdf<br><br>https://www.saica.co.za/Portals/0/Technical/LegalAndGovernance/37139.pdf<br><br>https://www.gov.za/sites/default/files/gcis_document/201409/a55-980.pdf<br><br>https://www.gov.za/sites/default/files/gcis_document/201409/37238act47of2013employequity.pdf<br><br>https://www.gov.za/sites/default/files/gcis_document/201409/act85of1993.pdf<br><br>https://www.gov.za/sites/default/file

[8] See the 2019 Policy Framework at 14.

	are:<br>• section 10 – "Everyone has the right to dignity and the right to have their dignity respected and protected";<br>• section 13 – "No one may be subjected to slavery, servitude or forced labour";<br>• section 22 – "Every citizen has the right to choose their trade, occupation or profession freely . . .";<br>• section 23 – "Everyone has the right to fair labour practices . . ."; and<br>• section 27 – "Everyone has the right to have access to— (a) healthcare services . . ."<br><br>The definitions of "employee" in the core labour legislation in South Africa (LRA, BCEA, EEA and the OHSA) include persons who "in any manner" assist with or further the business of an employer. Thus, all CHWs, including volunteers, are for the most part afforded the same protection as employees under this legislation.<br><br>A notable exception to this is the minimum wage which, though included as a "basic condition of employment" in the BCEA, applies only to "workers" as defined in the NMW Act.<br><br>The NMW Act entitles workers to payment of a wage in an amount no less than the national minimum wage, and every employer must pay to its workers, wages that are no less than that minimum.<br><br>A "worker" is defined as "any person who works for another and who receives, or is entitled to receive, any payment for that work whether in money or in kind." This means that CHWs legally entitled (through employment contracts or otherwise) to receive payment for their work will be entitled to at least the minimum wage. This would include the public sector and other workers covered by the Bargaining Council Resolution. The current minimum wage under Schedule 1 of the NMW Act is ZAR 20[9]/hour but this is subject to annual review.	s/gcis_document/201409/act181of1993.pdf
10.	**Are employment-related benefits (e.g. cost-of-living adjustments or pension scheme contributions) and employment requirements (e.g. vaccination) regulated or specified in contracts?**	http://www.dpsa.gov.za/dpsa2g/documents/pscbc/1999/03.pdf<br><br>Table of Provincial Programmes: https://www.spotlightnsp.co.za/wp-content/uploads/2016/11/CHW-per-

[9] Approximately US$1.09.

Legal Protections and Benefits for CHWs		
	Provision 3.3.3(b) of the Implementation Toolkit of the 2011 Provincial Guidelines recommend basic benefits in terms of the BCEA. The draft contract for CHWs in the Guidelines further states that the salary contains a portion in lieu of benefits, and that the general benefits shall be determined in terms of the Public Service Co-ordinating Bargaining Council Resolution 3 of 1999 and/or collective agreements in a relevant bargaining council. Annexure B of Public Service Co-ordinating Bargaining Council Resolution 3 of 1999 makes provision for a host of benefits including bonuses, incentive schemes and paid leave. In reality, providing for a portion "in lieu of benefits" means that the benefits are not practically taken into account in formulating salaries. There are no benefits provided for in the Bargaining Council Resolution.<br><br>The benefits given to CHWs vary depending on who the employer is. In practice, different provinces provide different benefits, such as a bonus and unpaid maternity leave. This is evident from the table of provincial programmes.	Province.pdf
